# Loneliness and low life satisfaction associated with older adults’ poor oral health

**DOI:** 10.3389/fpubh.2024.1428699

**Published:** 2024-08-09

**Authors:** T. L. Finlayson, K. L. Moss, J. A. Jones, J. S. Preisser, J. A. Weintraub

**Affiliations:** ^1^Health Management and Policy, San Diego State University School of Public Health, San Diego, CA, United States; ^2^Department of Biostatistics and Health Data Science, Adams School of Dentistry, University of North Carolina at Chapel Hill, Chapel Hill, NC, United States; ^3^University of Detroit Mercy, Detroit, MI, United States; ^4^Department of Biostatistics, Gillings School of Global Public Health, University of North Carolina at Chapel Hill, Chapel Hill, NC, United States; ^5^Department of Pediatric Dentistry and Dental Public Health, Adams School of Dentistry, University of North Carolina at Chapel Hill, Chapel Hill, NC, United States

**Keywords:** loneliness, life satisfaction, oral health, quality of life, latent class analysis, psychosocial, older adults

## Abstract

**Objective:**

To examine the association of older adults’ loneliness, life satisfaction, and other psychological stressors and resources with oral health status.

**Methods:**

This study merged 2018 data from the Health and Retirement Study (HRS) CORE survey with the HRS-Dental Module, and Psychosocial and Lifestyle Questionnaire–Panel A “Leave Behind” surveys (HRS-LB)(*N* = 418). Dental Module outcomes of interest were self-rated oral health status (SROH), and oral health-related quality of life (OHQOL). Older adults reported on loneliness, life satisfaction, perceived age, social status, control, mastery, and chronic stressors. Three distinct profiles based on the distribution of loneliness and life satisfaction were previously identified in the combined HRS and HRS-LB study population (*N* = 4,703) using latent class analysis (LCA). Class A:“Not Lonely/Satisfied” adults had the fewest psychosocial risk factors and most resources; Class C:“Lonely/Unsatisfied” adults exhibited the opposite profile (most risk factors, fewest resources); Class B:“Lonely/Satisfied” adults exhibited loneliness with favorable life satisfaction. Regression models examined associations between LCA classes and fair/poor SROH and the OHQOL scale score and individual items, after adjusting for socio-demographics.

**Results:**

About 13% of older adults experienced loneliness, and about 16% reported low life satisfaction. About one-quarter (28%) of older adults reported fair/poor SROH, and they experienced more psychosocial risk factors than their counterparts with better oral health status. Nearly half the older adults were categorized in Class A:“Not Lonely/Satisfied” (*n* = 201), and about one-quarter each in Class B:“Lonely/Satisfied” (*n* = 103) and Class C:“Lonely/Unsatisfied” (*n* = 112). In fully adjusted models, Class B older adults had 1.81 (1.11–2.96) times greater odds of fair/poor SROH, and Class C had 4.64 (2.78–7.73) times greater odds of fair/poor SROH than Class A. Fully adjusted linear regression model results indicated a gradient by LCA class. OHQOL varied; Class A older adults had the best (lowest) OHQOL score (mean = 8.22, 4.37–12.10), Class B scored in the middle (mean = 12.00, 7.61–16.50), while Class C had the worst (highest) OHQOL score (mean = 16.20, 11.80–20.60).

**Conclusion:**

Loneliness, as a defining characteristic distinguishing three latent classes of older adults, was associated with more risk factors and poorer oral health outcomes. Loneliness, life satisfaction, perceived age, social status, control, mastery, and chronic stressors vary widely for older adults and matter for oral health and OHQOL.

## 1 Introduction

Loneliness and social isolation have been recently recognized in the United States (US) by the National Academies of Sciences, Engineering and Medicine (1) as important social determinants of health that can potentially negatively affect health and quality of life among older adults. Loneliness is a subjective feeling, that may result from experiencing social isolation, which is the objective and measurable lack of connection and interaction with other people (1). Social relationships and connections can vary widely, in terms of frequency and quality of interactions. Social isolation is a potential precursor to loneliness if the frequency and quality are perceived to be insufficient, but they are distinct, despite often being referred to in tandem. Loneliness may or may not occur in socially isolated individuals, and can be experienced either temporarily, or as a more permanent undesirable state of being over time (2).

Older adults may be especially at-risk for experiencing loneliness, given life changes occurring with aging, such as retirement from the workforce and disabilities that may limit their ability to regularly socially interact with and feel emotionally connected to other people. The demographic composition of the US is getting older, and Americans are living longer (3). Recent analyses suggest that older adults are not any more lonely compared to the prior decade, though there will be more older Americans (4). In a recent meta-analysis, the negative impact of loneliness had an estimated 26% increased likelihood of mortality (5). Loneliness has also been linked to poor health outcomes (6, 7), incident stroke (8), and lower quality of life among older adults ((9, 10)), but less is known about the potential impact of loneliness on oral health outcomes and oral health-related quality of life (OHQOL).

Among older adults in India, being lonely and having more disabilities were each associated with a greater number of decayed, missing or filled teeth, worse periodontal disease status, and edentulism (missing all natural teeth) (11). In a cross-sectional study of Japanese older adults, loneliness was associated with having fewer than 20 teeth, and both loneliness and social isolation were associated with less ability to chew foods (12). In a longitudinal study of Chinese older adults, social isolation but not loneliness was associated with fewer remaining teeth and an accelerated rate of tooth loss between 2011 and 2018 (13). There are many reasons why loneliness, life satisfaction and poor oral health can be connected, and these associations can potentially go in both directions. One sex-stratified longitudinal study of older adults in Germany found that both men and women were more lonely if their overall self-rated health decreased, and women were more lonely if they postponed dental visits due to cost and had fewer chronic diseases (14). People with poor oral health may have toothaches, be in chronic pain, and have difficulty eating, chewing and communicating. They may be embarrassed by poor dental and facial esthetics from stained, broken or missing teeth or their replacements, have poor-fitting or uncomfortable dentures, or poor occlusion. Consequently, they may avoid eating and socializing with others, leading to loneliness and social isolation (15).

Self-reported oral health outcomes, like self-rated oral health (SROH) status and OHQOL, are meaningful indicators of overall oral health. SROH is a useful measure when clinical assessments are not available, and correlate well with clinically-defined oral health status (16, 17). There is also a growing body of evidence exploring how psychological factors relate to oral health. Among Australian adults, self-efficacy (beliefs about one’s own ability to engage in certain health behaviors) was positively associated with better self-rated oral health and better OHQOL, accounting for perceived stress, ability to cope, and fatalistic beliefs (18). In a study examining the role of psychosocial factors on oral health of adults in Norway, sense of coherence (a perception about one’s global ability and resources) was found to be linked with other resources that facilitated use of dental services and fewer dental needs (19). In a US national cross-sectional dataset with many psychological measures, chronic stress was associated with fair/poor SROH among adults, while psychosocial resources (mastery, self-esteem) were protective (20). In a longitudinal analysis of the US Health and Retirement Study (HRS), a nationally representative sample of US older adults over age 50, Tembhe et al. (21) found that about 26% of older adults had worse SROH at both timepoints in 2008 and 2018. Older adults with better SROH tended to have higher socioeconomic status and better access to dental care.

Poor OHQOL and loneliness and low life satisfaction have been linked. In a study of older adults in England, Rouxel et al. (22) found an association with poorer OHQOL and increased odds of loneliness. Following older adults over time, those with new oral concerns with negative impact on function were also more likely to become lonely. Life satisfaction has also been associated with general quality of life and OHQOL in other countries (23–25). A recent cross-sectional study of older adults in Mexico used a latent class analysis (LCA) approach to examine several clinically-assessed oral health status indicators and OHQOL (26). The researchers identified three LCA classes, and found LCA useful to discriminate between groups by oral health status and show older adults with poorer oral health had poorer OHQOL scores. The LCA approach maximizes homogeneity within classes, by grouping individuals together who respond in a similar way. LCA approaches to health research provide insights into patterns of risk profiles (27), and are particularly useful for exploring multi-dimensional constructs like OHQOL. They could assist clinicians with identification of those who are lonely for timely interventions.

The purpose of this paper is to estimate the association of older adults’ experiences with loneliness, life satisfaction, and other psychological stressors and resources with SROH and OHQOL using 2018 US HRS data. Three distinct profiles of adults based on the distribution of loneliness and life satisfaction were previously identified in the combined HRS and HRS-LB study population (*N* = 4,703) using LCA (28). Class A:“Not Lonely/Satisfied” adults had the fewest psychosocial risk factors and most resources; Class C:“Lonely/Unsatisfied” adults exhibited the opposite profile (most risk factors, fewest resources); Class B:“Lonely/Satisfied” adults exhibited loneliness with favorable life satisfaction. Regression models examined associations between the LCA classes and SROH and the OHQOL scale score and individual items, after adjusting for socio-demographics in the subset of HRS older adults (approximately 10%) who participated in the HRS-Dental Module.

## 2 Materials and methods

### 2.1 Study design

This cross-sectional analysis examined psychosocial factors and dental outcomes among older adults that participated in the HRS, led by the University of Michigan. This secondary data analysis of publicly available and de-identified data did not require ethics approval from the University of North Carolina at Chapel Hill.

### 2.2 Data sources

The full US HRS dataset includes about 20,000 older adults (>50 years old), which comprise a nationally representative sample (29, 30); the present analysis utilizes a subset of HRS data. Data from the 2018 HRS-CORE survey were linked with 2018 HRS “Leave Back” (HRS-LB) Subsample A survey and the 2018 HRS-Dental Module to create the final analytic sample who were not missing data (*N* = 416). The biennial HRS-CORE survey was conducted via face-to-face and telephone interviews with the full HRS sample. Select socio-demographic characteristics were drawn from the HRS-CORE for this analysis. The HRS-LB survey is left with participants to complete and mail back. It included many validated psychosocial scales across six domains to capture overall well-being, lifestyle, self-related beliefs, work, social relations/support, and personality traits (31). The first three domains (well-being, lifestyle, and self-related beliefs) were selected for this analysis. These three domains encompassed many established psychosocial variables of interest. The HRS-LB has two subsamples from the enhanced face-to-face interviews, and is asked every 4 years, from approximately one-half of HRS-CORE participants. The 2018 HRS-Dental Module, or HRS “experimental module” that is not asked regularly, selected 10% of HRS-CORE participants, similar to other HRS experimental modules. However, while those other modules were based on random samples of HRS-CORE participants, the 2018 Dental Module was completed by a convenience subsample of HRS-CORE participants as efforts were made to include participants from the 2008 HRS-Dental Module. The longitudinal data analysis of Tembhe et al. (21) utilized the overlap of the 2008 and 2018 dental modules (while excluding HRS-LB survey data), whereas the analysis in this article pertains to a larger amount of variables in a cross-sectional 2018 HRS dataset created by merging the three aforementioned HRS surveys.

### 2.3 Variables

#### 2.3.1 Outcome measures

Two oral health measures were in the 2018 HRS-Dental Module. Self-reported oral health status (SROH) was assessed as Excellent, Very Good, Good, Fair, or Poor, then dichotomized as Fair/Poor vs. Excellent/Very Good/Good (32). The Oral Health Quality of Life (OHQOL) short-form was the sum of 5 items (plus a sixth item, if the respondent had dentures); items described avoiding eating some foods, finding it difficult to relax, and avoiding going out, or uncomfortable dentures because of problems with teeth or dentures. Possible responses were never, hardly ever, occasionally, fairly often, or very often. Further, participants were asked if they were nervous or self-conscious because of problems with their teeth or dentures (responses were never, sometimes, or always) and how much pain they had from their teeth or dentures, with possible responses: none at all, a little bit, some, quite a bit, or a great deal (33). Scores were rescaled (0–100), with higher OHQOL scores being worse, reflecting poorer quality of life due to oral problems.

#### 2.3.2 HRS-LB psychosocial variables

HRS-LB scale scoring instructions were followed (31). Individual items for HRS-LB scales were also dichotomized for inclusion in the latent class analysis (LCA). Individual items for HRS-LB scales were dichotomized as some response categories for the individual psychosocial variables had small cell sizes. The benefit of dichotomization of multi-category variables was to mitigate computational challenges by reducing the complexity (i.e., dimension of the parameter space) of LCA models.

Loneliness was measured with the University of California Los Angeles (UCLA) 11-item measure (34–36), reflecting on how often individuals felt lonely (hardly ever/never, sometimes, or often). This is a valid and frequently used measure for loneliness. More frequent loneliness is captured by higher scale scores; for LCA, this was dichotomized as hardly ever/never vs. sometimes/often.

The Life Satisfaction Scale captured respondents’ agreement with a series of statements, including “I am satisfied with my life” on a 7-point Likert scale (1 = strongly disagree, 7 = strongly agree). Lower scores indicate less satisfaction (37). The dichotomized version collapsed the disagree categories and the neutral/agree categories.

The life-situation specific satisfaction 7-item scale measured health, family life, financials and living situation. Items included rating satisfaction with “your health” and “daily life and leisure activities,” with 1 = completely and 5 = not at all satisfied. Lower scores indicate less satisfaction (38). The dichotomized version collapsed completely and very satisfied categories versus the rest. Collectively, these three sets of measures assessed the “well-being” domain.

In the “beliefs domain,” respondents reported perceptions about their age, social status and how that has changed in recent years, and levels of control, mastery, and self-efficacy. A single question asked: “Many people feel older or younger than they actually are. What age do you feel?” Participant reported whether or not they felt older than they actually were, to operationalize perceived age as a potential risk factor. Participants completed an aging 8-item scale about feelings about getting older, with items like “Things keep getting worse as I get older” (39, 40). Responses were 1 = strongly disagree to 6 = strongly agree, and items were averaged after appropriate reverse coding, with lower scores indicating lower satisfaction with perceived aging. Dichotomies collapsed disagree and agree categories.

Subjective social status included two questions with reference to placement and movement in the last 2 years on a 10-step social ladder Status (41, 42). The 10-steps got split at ≥7 to indicate high social status. We dichotomized moving down versus improvement/no change.

Sense of control has 10-items total, half focused on constraints, and half on mastery, and are scored as two separate constructs using the same 6-point scale (1 = strongly disagree, 6 = strongly agree) (43). Higher scores indicated more constraints and higher levels of mastery, respectively. More constraints are a negative risk factor, while more mastery is a positive resource factor. Disagree and agree categories were collapsed for dichotomization.

Control over health, social life, and financial situation were each single items on a 0–10 scale (0 = no, 10 = very much control) (44). These were each dichotomized at 7+, indicating high control/perceived efficacy.

“Lifestyle” domain catalogued the presence and effect of eight stressors over the past year, like housing problems (45). We coded if participants rated each stressor as occurring and somewhat/very upsetting.

#### 2.3.3 HRS-CORE variables

HRS defines birth cohorts as: Asset and Health Dynamics Among the Oldest Old (AHEAD cohort, born before 1924); Children of the Depression (CODA cohort, born 1924–1930); Original HRS cohort (born 1931–1941); War Baby cohort (born 1942–1947); and Early/Middle/Late Baby Boomer cohorts (born 1948–1965). Given small cell sizes, we combined AHEAD/CODA cohorts. Four birth cohorts were included in our analyses. Race/ethnicity, sex, education, marital status, household net wealth, Medicaid participation, urban residency, current smoker, current drinker, and diabetes were included as covariates.

### 2.4 Statistical analyses

As we conducted a complete case analysis, our final analytic dataset had no missingness across variables. Descriptive statistics were computed for all variables. Psychosocial scales were scored to examine distributions across the sample overall, and by outcomes of interest. Participants were included in one of three classes based on the highest posterior probability of membership as determined in the previously conducted analysis of the much larger dataset from combined 2018 HRS-Core and HRS-LB surveys (28). Details about the distribution of individual psychosocial variables and summary of how each was dichotomized are reported in the supplemental materials file (28). A heatmap was created to provide a graphical representation of the distribution of responses to the psychosocial scales, to illustrate the differences across the three LCA classes.

Logistic regression models were fitted to estimate the association of the latent classes (Class A as reference) for the dichotomous SROH outcome. Odds ratios and 95% confidence intervals (CI) were computed. Multiple linear regression models were fitted to estimate the association of the latent classes with mean OHQOL score and component items. Minimally-adjusted models included LCA class and fixed demographics (race, sex, birth cohort). Full models adjusted for education, marital status, wealth, Medicaid, urban, smoking, alcohol, and diabetes. SAS software version 9.4 was used for all analyses (SAS Institute, Cary, NC).

## 3 Results

**Figure 1:**
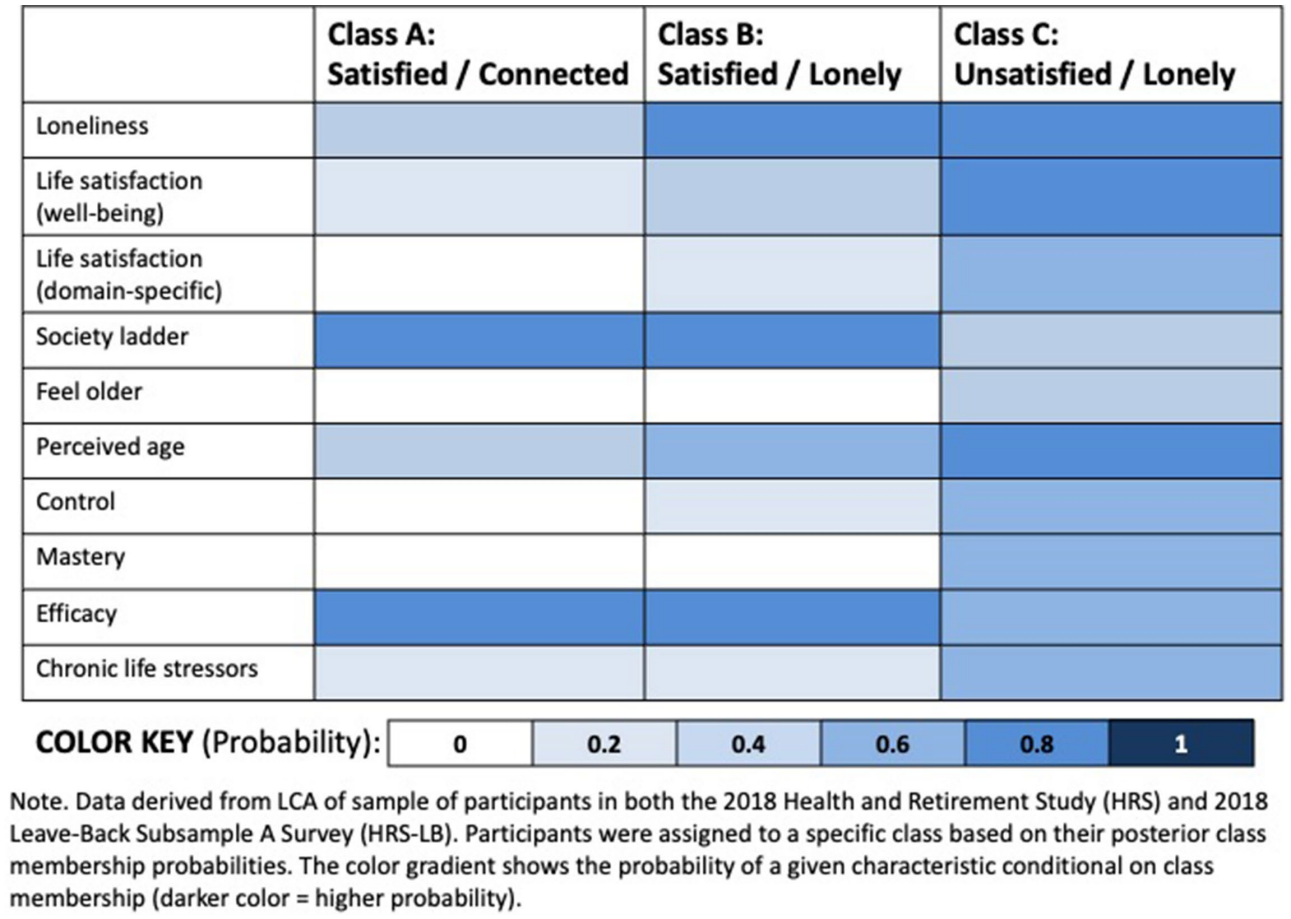
Heatmap of Latent Class Analysis (LCA) of psychosocial characteristics among older adults in the US (*n* = 4,703). Data derived from LCA of sample of participants in both the 2018 Health and Retirement Study (HRS) and 2018 Leave-Back Subsample a Survey (HRS-LB). Participants were assigned to a specific class based on their posterior class membership probabilities. The color gradient shows the probability of a given characteristic conditional on class membership (darker color = higher probability).

In the current analysis of HRS participants who participated in the 2018 Dental-Module, the three LCA classes (Class A: “Not Lonely/Satisfied,” Class B: “Lonely/Satisfied” or Class C: “Lonely/Unsatisfied”) each exhibited different psychosocial profiles with lower, moderate or higher risk factors, respectively (Figure 1: Heatmap).

Table 1 summarizes the distribution of demographic and psychosocial characteristics for HRS-Dental Module participants overall, and by SROH categories and OHQOL mean score. All demographic characteristics except sex, birth cohort, and urban residency notably varied by oral health outcomes. Psychosocial characteristics were distributed in the expected direction; among older adults who reported fair/poor SROH (28%), they were worse off than their counterparts with better (excellent/very good/good) SROH: they were lonely more frequently, less satisfied with life, felt negatively about aging and older than their chronological age, reported less mastery and more constraints, downward movement on the social ladder, less control, and more stressors that were upsetting. For older adults with worse OHQOL (higher scores), the patterns were similar to the adults with fair/poor SROH. The overall mean OHQOL score was 11.4, with a standard deviation of 17.7.

**Table 1:** Demographic and psychosocial characteristics, by self-rated oral health status and oral health quality of life (OHQOL), HRS 2018 dental module sample (*n* = 416).

	*N* = 416	Excellent or very good or good *N* = 301	Fair or poor *N* = 115	Mean (SD) OHQOL^3^
DEMOGRAPHICS	*N* (%)	*N* (%)	*N* (%)	Mean (SD)
Race				
Caucasian	292 (70.9%)	226 (75.8%)	66 (57.9%)	9.22 (15.7)
African American	51 (12.4%)	27 (9.1%)	24 (21.1%)	18.0 (21.4)
Hispanic	44 (10.7%)	25 (8.4%)	19 (16.7%)	18.0 (22.5)
Other	25 (6.1%)	20 (6.7%)	5 (4.4%)	13.7 (17.0)
Sex				
Female	250 (60.1%)	183 (60.8%)	67 (58.3%)	12.0 (18.2)
Male	166 (39.9%)	118 (39.2%)	48 (41.7%)	10.5 (16.8)
Birth Cohort				
AHEAD & CODA	13 (3.1%)	11 (3.7%)	2 (1.7%)	7.1 (12.0)
HRS Original	88 (21.2%)	60 (19.9%)	28 (24.4%)	13.1 (17.8)
War Babies	59 (14.2%)	41 (13.6%)	18 (15.7%)	9.0 (11.3)
Baby Boomers	256 (61.5%)	189 (62.8%)	67 (58.3%)	11.6 (19.0)
Education				
< High School	46 (11.1%)	24 (8.0%)	22 (19.1%)	21.4 (21.8)
High School or Equivalent	245 (58.9%)	166 (55.2%)	79 (68.7%)	13.0 (19.1)
College +	125 (30.1%)	111 (36.9%)	14 (12.2%)	4.7 (8.4)
Marital Status				
Married	247 (59.7%)	189 (63.0%)	56 (49.1%)	8.8 (14.6)
Not Married	167 (40.3%)	111 (37.0%)	58 (50.9%)	15.2 (20.8)
Live Alone				
Yes	87 (20.9%)	62 (20.6%)	25 (21.7%)	12.5 (20.0)
No	329 (79.1%)	239 (79.4%)	90 (78.3%)	11.1 (17.0)
Household Net Wealth				
<$50,000 - $50,000	107 (25.7%)	59 (19.6%)	48 (41.7%)	22.7 (24.5)
>$50,000 - $200,000	81 (19.5%)	52 (17.3%)	29 (25.2%)	9.9 (13.2)
>$200,000 - $500,000	91 (21.9%)	77 (25.6%)	14 (12.2%)	7.0 (11.9)
>$500,000	137 (32.9%)	113 (37.5%)	24 (20.9%)	6.4 (12.2)
Medicaid				
Yes	52 (12.5%)	28 (9.3%)	24 (20.9%)	25.3 (27.0)
No	363 (87.5%)	272 (90.7%)	91 (79.1%)	9.5 (14.9)
Location				
Urban	214 (51.8%)	154 (51.5%)	60 (52.6%)	11.8 (18.5)
Suburban	91 (22.0%)	70 (23.4%)	21 (18.4%)	12.6 (19.8)
Ex-urban	108 (26.2%)	75 (25.1%)	33 (29.0%)	9.5 (13.6)
Current Smoker				
Yes	46 (11.1%)	23 (7.7%)	23 (20.0%)	24.1 (21.6)
No	368 (88.9%)	276 (92.3%)	92 (80.0%)	9.9 (16.5)
Current Drinker				
Yes	249 (60.3%)	195 (65.4%)	54 (47.0%)	14.9 (20.2)
No	164 (39.7%)	103 (34.6%)	61 (53.0%)	9.2 (15.5)
Diabetes				
Yes	110 (26.6%)	66 (22.1%)	44 (38.6%)	16.1 (21.3)
No	303 (73.4%)	233 (77.9%)	70 (61.4%)	9.7 (15.9)

Psychosocial characteristics	Mean (SD)			
Lonely Scale, Mean(SD)^**1**^	1.52 (0.44)	1.46 (0.41)	1.69 (0.48)	0.30^4^
Life Satisfaction Wellbeing Scale, Mean(SD)^**2**^	5.10 (1.59)	5.35 (1.47)	4.46 (1.70)	−0.32^4^
Life satisfaction domain specific scale, mean (SD)^**d**^	3.67 (0.77)	3.81 (0.70)	3.29 (0.81)	−0.32^4^
Perceived Age Scale, Mean(SD)^**2**^	3.99 (1.04)	4.13 (0.99)	3.62 (1.10)	-0.28^4^
Feel Older				
Yes	51 (12.6%)	31 (10.4%)	20 (18.7%)	18.3 (19.1)
No	354 (87.4)	267 (89.6%)	87 (81.3%)	10.5 (17.4)
Constraints Scale, Mean(SD)^**1**^	2.06 (1.13)	1.85 (0.99)	2.63 (1.27)	0.25^4^
Mastery Scale, Mean(SD)^**2**^	4.78 (1.14)	4.87 (1.15)	4.55 (1.09)	-0.09^4^
Perceived Change in Social Status, Mean(SD)^**2**^	6.63 (1.79)	7.00 (1.56)	5.63 (1.99)	-0.28^4^
Moved in Social Status				
Up	72 (17.5%)	53 (17.8%)	19 (16.7%)	9.62 (14.8)
Down	35 (8.5%)	21 (7.1%)	14 (12.3%)	19.0 (21.3)
No Change	305 (74.0%)	224 (75.2%)	81 (71.1%)	11.0 (17.7)
Control Domain^**2**^				
Over Health	7.70 (2.07)	7.94 (1.87)	7.08 (2.40)	-0.17^4^
Over Social Life	8.20 (2.01)	8.38 (1.82)	7.72 (2.39)	-0.22^4^
Over Financial Situation	7.66 (2.33)	7.94 (2.13)	6.94 (2.64)	-0.12^4^
Lifestyle (% Upsetting)				
Self-Health Problems	150 (36.1%)	92 (30.6%)	58 (50.4%)	17.3 (19.8)
Phy/Emot Problems in SP/Child	135 (32.5%)	96 (31.9%)	39 (33.9%)	14.8 (20.1)
Drug/Alcohol Probs Fam Member	52 (12.5%)	31 (10.3%)	21 (18.3%)	22.3 (23.1)
Financial Strain	92 (22.1%)	54 (17.9%)	38 (33.0%)	18.4 (22.3)
Housing Problems	30 (7.2%)	17 (5.7%)	13 (11.3%)	23.5 (19.7)
Problems in Relationship	64 (15.4%)	48 (16.0%)	16 (13.9%)	12.8 (17.5)
Reg Help Ailing Friend/Fam	49 (11.8%)	31 (10.3%)	18 (15.7%)	13.9 (20.6)

1 Higher mean scores are worse (higher psychosocial risk) for these scales: loneliness, constraints.

2 Higher mean scores are better (lower psychosocial risk; more psychosocial resources) for these scales: life satisfaction wellbeing, life satisfaction domain-specific, perceived age, mastery, change in social status, and control.

3 OHQOL, oral health quality of life summary score, higher scores indicate worse OHQOL. OHQOL includes items related to avoid foods, difficult to relax, avoided going out, self-conscious, and pain.

4 Pearson correlation.

Table 2 shows the HRS-Dental Module LCA distribution. Nearly half the sample were in Class A (*n* = 201), about one-quarter each were in Classes B and C (n = 103 and 112, respectively). The three LCA class profiles and distributions of sociodemographic and psychosocial characteristics followed patterns. The lowest risk class (Class A:“Not Lonely/Satisfied”) had the most psychosocial resources and fewest risk factors, while the converse was true for Class C:“Lonely/Unsatisfied” older adults. Class B:“Lonely/Satisfied” generally fell in between Class A and C for most psychosocial characteristics, though loneliness emerged as a prominent risk factor (closer to Class C loneliness scores), while life satisfaction scores more closely mirrored Class A.

**Table 2:** Mean (SE) scale items by LCA Class, HRS 2018 dental module sample (*n* = 416).

	Class A: Not Lonely/ Satisfied *N* = 201	Class B: Lonely/ Satisfied *N* = 103	Class C: Lonely/ Unsatisfied *N* = 112	*p*-value
**Demographics**				
Race				0.02
Caucasian	152 (76.4%)	69 (67.7%)	71 (64.0%)	
African American	24 (12.1%)	11 (10.8%)	16 (14.4%)	
Hispanic	14 (7.0%)	18 (17.7%)	12 (10.8%)	
Other	9 (4.5%)	4 (3.9%)	12 (10.8%)	
Sex				0.02
Female	132 (65.7%)	50 (48.5%)	68 (60.7%)	
Male	69 (34.3%)	53 (51.5%)	44 (39.3%)	
Birth Cohort				0.07
AHEAD & CODA	9 (4.5%)	3 (2.9%)	1 (0.9%)	
HRS Original	41 (20.4%)	25 (24.3%)	22 (19.6%)	
War Babies	38 (18.9%)	9 (8.7%)	12 (10.7%)	
Baby Boomers	113 (56.2%)	66 (64.1%)	77 (68.8%)	
Education				0.03
< High School	22 (11.0%)	11 (10.7%)	13 (11.6%)	
High School or Equivalent	105 (52.2%)	63 (61.2%)	77 (68.8%)	
College +	74 (36.8%)	29 (28.2%)	22 (19.6%)	
Marital Status				0.006
Married	131 (65.8%)	63 (61.2%)	53 (47.3%)	
Not Married	68 (34.2%)	40 (38.8%)	59 (52.7%)	
Live Alone				0.049
Yes	39 (19.4%)	16 (15.5%)	32 (28.6%)	
No	162 (80.6%)	87 (84.5%)	80 (71.4%)	
Household Net Wealth				<0.0001
<$50,000 - $50,000	41 (20.4%)	22 (21.4%)	44 (39.3%)	
>$50,000 - $200,000	34 (16.9%)	17 (16.5%)	30 (26.8%)	
>$200,000 - $500,000	47 (23.4%)	23 (22.3%)	21 (18.8%)	
>$500,000	79 (39.3%)	41 (39.8%)	17 (15.2%)	
Medicaid				0.36
Yes	21 (10.5%)	13 (12.6%)	18 (16.1%)	
No	179 (89.5%)	90 (87.4%)	94 (83.9%)	
Location				0.79
Urban	107 (53.8%)	53 (51.5%)	54 (48.7%)	
Suburban	44 (22.1%)	24 (23.3%)	23 (20.7%)	
Ex-urban	48 (24.1%)	26 (25.2%)	34 (30.6%)	
Current Smoker				0.009
Yes	16 (8.0%)	9 (8.8%)	21 (18.9%)	
No	185 (92.0%)	93 (91.2%)	90 (81.1%)	
Current Drinker				0.10
Yes	130 (65.7%)	58 (56.3%)	61 (54.5%)	
No	68 (34.3%)	45 (43.7%)	51 (45.4%)	
Diabetes				0.20
Yes	46 (23.1%)	28 (27.2%)	36 (32.4%)	
No	153 (76.9%)	75 (72.8%)	75 (67.6%)	
**Psychosocial characteristics**				
Lonely Scale, Mean(SD)^**1**^	1.17 (0.16)	1.77 (1.77)	1.91 (0.43)	<0.0001
Life Satisfaction Scale, Mean(SD)^**2**^	5.94 (1.15)	5.36 (1.05)	3.35 (1.26)	<0.0001
Life satisfaction domain-specific scale, mean (SD)^**2**^	4.01 (0.57)	3.63 (0.57)	2.92 (0.63)	<0.0001
Perceived Age Scale, Mean(SD)^**2**^	4.54 (0.85)	3.90 (0.78)	3.08 (0.90)	<0.0001
Feel Older				
Yes	7 (3.6%)	9 (8.7%)	35 (32.4%)	
No	187 (96.4%)	94 (91.3%)	73 (67.6%)	<0.0001
Constraints Scale, Mean(SD)^**1**^	1.51 (0.78)	2.15 (1.02)	2.97 (1.15)	<0.0001
Mastery Scale, Mean(SD)^**2**^	5.12 (1.05)	4.95 (0.86)	4.01 (1.16)	<0.0001
Perceived Change in Social Status, Mean(SD)^**2**^	7.31 (1.38)	6.86 (1.68)	5.20 (1.75)	<0.0001
Moved in Social Status				<0.0001
Up	38 (19.1%)	24 (23.5%)	10 (9.0%)	
Down	4 (2.0%)	3 (2.9%)	28 (25.2%)	
No Change	157 (78.9%)	75 (73.5%)	73 (65.8%)	
Control Domain, Mean (SD)^**2**^				
Over Health	8.18 (1.66)	8.03 (1.73)	6.55 (2.52)	<0.0001
Over Social Life	9.03 (1.18)	8.07 (1.89)	6.81 (2.48)	<0.0001
Over Financial Situation	8.48 (1.62)	8.03 (1.84)	5.86 (2.79)	<0.0001
Lifestyle (% Upsetting)				
Self-Health Problems	41 (20.4%)	32 (31.1%)	77 (68.8%)	<0.0001
Phy/Emot Problems in SP/Child	42 (20.9%)	28 (27.2%)	65 (58.0%)	<0.0001
Drug/Alcohol Probs Fam Member	12 (6.0%)	13 (12.6%)	27 (24.1%)	<0.0001
Financial Strain	11 (5.5%)	10 (9.7%)	71 (63.4%)	<0.0001
Housing Problems	4 (2.0%)	3 (2.9%)	23 (20.5%)	<0.0001
Problems in Relationship	10 (5.0%)	14 (13.6%)	40 (35.7%)	<0.0001
Reg Help Ailing Friend/Fam	14 (7.0%)	8 (7.8%)	27 (24.1%)	<0.0001

1 Higher mean scores are worse (higher psychosocial risk) for these scales: loneliness, constraints.

2 Higher mean scores are better (lower psychosocial risk; more psychosocial resources) for these scales: life satisfaction wellbeing, life satisfaction domain-specific, perceived age, mastery, change in social status, and control.

Table 3 shows the minimally and fully adjusted odds ratios for the two dichotomous outcomes comparing LCA classes. In fully adjusted models, Class B older adults had 1.81 (1.11–2.96) times greater odds to have fair/poor SROH than not lonely Class A. Similarly, Class C older adults, the lonelier and less satisfied with life group, had 4.64 (2.78–7.73) times greater odds of fair/poor SROH. Odds ratio estimates were attenuated in the fully adjusted models over the minimally adjusted models, but overall, older adults in both Classes B and C remained statistically significantly more likely to experience poor oral health outcomes than Class A.

**Table 3:** Adjusted estimates (CI) for comparison of dental outcomes by LCA Class, 2018 (*N* = 416).

	Minimally Adjusted^1^			Fully Adjusted^2^		
Outcome variable	Class A: Not Lonely/ Satisfied	Class B: Lonely/Satisfied	Class C: Lonely/ Unsatisfied	Class A: Not Lonely/ Satisfied	Class B: Lonely/ Satisfied	Class C: Lonely/ Unsatisfied
		Odds ratio			Odds ratio	
Fair/Poor Self-Rated Oral Health (SROH)^3^	Ref	1.83 (1.15–2.91)	5.20 (3.24–8.36)	Ref	1.81 (1.11–2.96)	4.64 (2.78–7.73)
		Difference in means			Difference in means	
Mean (95% CI) OHQOL^4^^,^^5^	10.2 (6.32–14.3)	14.7 (10.1–19.3)	21.5 (17.1–26.0)	8.22 (4.37–12.1)	12.0 (7.61–16.5)	16.2 (11.8–20.6)
OHQOL Items		Odds ratio			Odds ratio	
Avoid Foods^6^	Ref	1.53 (0.80–2.93)	2.82 (1.57–5.08)	Ref	1.39 (0.67–2.87)	1.83 (0.94–3.60)
Difficult To Relax^6^	Ref	2.21 (0.94–5.21)	4.89 (2.29–10.5)	Ref	1.91 (0.77–4.74)	3.41 (1.47–7.91)
Avoided Going Out^6^	Ref	1.71 (0.47–6.27)	2.33 (0.72–7.51)	Ref	1.73 (0.37–8.05)	2.28 (0.52–10.0)
Self-Conscious^6^	Ref	1.25 (0.58–2.70)	3.76 (2.00–7.04)	Ref	1.12 (0.47–2.65)	2.96 (1.45–6.03)
Pain^6^	Ref	2.18 (0.99–4.80)	3.83 (1.89–7.73)	Ref	2.13 (0.90–5.05)	3.56 (1.61–7.89)

1 Minimally Adjusted by Race, Sex and Birth Cohort.

2 Fully Adjusted by Race, Sex, Birth Cohort, Education, Marital Status, Wealth, Medicaid, Urban, Smoker, Alcohol Drinker, and Diabetes.

3 Covariate-adjusted odds ratio estimates are based on ordinary logistic regression.

4 Covariate-adjusted difference in means estimates are based on multiple linear regression.

5 OHQOL = oral health quality of life summary score, higher scores indicate worse OHQOL. OHQOL includes items related to avoid foods, difficult to relax, avoided going out, self-conscious, and pain.

6 Covariate-adjusted odds ratio estimates are based on cumulative logits logistic regression with the proportional odds assumption for three-category ordinal outcomes for the OHQOL items.

Results from the linear regression model indicate a clear gradient by LCA class for mean OHQOL scores. Class A older adults had the best (lowest) OHQOL in the minimally and fully adjusted models (means 10.2 and 8.22, respectively). Class B older adults had OHQOL scores in the middle (means 14.7 and 12.0, respectively), while older adults in Class C had the worst OHQOL with the highest scores (means 21.5 and 16.2, respectively). The individual OHQOL items followed similar patterns and Class C exhibited worse OHQOL than Class B in both the minimally and fully adjusted models.

## 4 Discussion

LCA results identified clear risk profiles and important relationships between loneliness, low life satisfaction, other psychosocial factors and two oral health outcomes. Class C, defined by loneliness and low life satisfaction, had the worst SROH status and OHQOL. The classes each had risk profiles in the expected directions, and the pattern of associations in our results are similar to findings from studies analyzing related variables in longitudinal HRS analyses. Among older adults who experienced positive changes in life satisfaction over 4 years, those with higher life satisfaction were less lonely, and fared better on a range of psychosocial measures of well-being and physical health outcomes and behaviors (46). Similarly, in HRS analyses among older adults over a four-year period found that those who were more satisfied with the aging process experienced better outcomes, across 35 different outcomes, including self-rated general health and many physical, behavioral and psychosocial outcomes (47). Our study adds oral health outcomes to this growing research area.

More psychosocial stressors and fewer resources were associated with worse self-rated oral health status and OHQOL. The three latent classes we identified may provide insights into patterns of risk profiles that may be helpful for clinicians. Additionally, the patterns of our LCA results also align with correlates of loneliness identified in a recent review; loneliness among older adults was associated with poor self-reported general health and a range of psychosocial risk factors, including low efficacy and negative life events (48). Our results also align with the results of a recent systematic review and meta-analysis, which identified five studies on oral health and loneliness, and found associations between lonely adults and a range of worse self-reported and clinical oral health outcomes (49). Our study also appears to be the first analysis on the topic of loneliness and oral health from the US (49).

We found that lonely older adults, whether they were satisfied with life or not, had worse oral health outcomes. Loneliness is potentially modifiable. In a review of 33 interventions intended to reduce loneliness among older adults between 1996 and 2011, there were several effective individual and group interventions identified for community-based delivery or in institutional settings (50). Many effective interventions used different types of technology. There was also strong potential in reducing loneliness through some group educational programs and shared activities programs, especially when attention was paid to addressing some psychosocial components to foster meaningful social connections. Psychosocial factors matter, since loneliness is the perception that existing social connections are not adequate. A higher proportion of Class C older adults who were lonely and unsatisfied identified problems in relationships as an upsetting chronic stressor. Enhancing social interactions may not address the feelings of loneliness for this group. Initially identifying individuals who are truly lonely and also open to any intervention may be difficult, and may not be needed; Class B older adults were lonely, but were also satisfied with life. Lonely and unsatisfied older adults in Class C experienced more chronic life stressors that were upsetting, many of which are more challenging in nature and less amendable to easily address, like financial strains. Psychosocial resources to counteract the negative impact of stressors may not be enough for chronic, on-going stressors if there is no way to address the source of the stressor. Many in Class C also noted their own health problems and problems with other family members as upsetting chronic stressors. These perceptions may reflect the reality that their health is poor, and while there may be interventions to slow deterioration and morbidity, there may not be effective ways to truly ameliorate declining health conditions.

Oral health is connected to overall health and perceptions about quality of life. In a recent systematic review examining how oral health factors affect OHQOL among older adults, OHQOL was better for those with more functional dentition (i.e., more teeth, more occluding pairs of teeth for chewing, appropriate prosthetics) (51). One way to potentially improve OHQOL for older adults is to ensure access to regular dental care to maintain the health and function of their natural teeth longer, or facilitate access to prosthetics like implants or dentures if needed. Cost is a common barrier to dental care, especially among this age group, that is often retiring and may have less income. In a recent meta-analysis of OHQOL, a social gradient relationship was found (52); no matter which measures of socioeconomic status (SES) and OHQOL were used, there were consistent findings with low SES corresponding to poor OHQOL. When older adults transition out of the workforce, they tend to live on a fixed income and changes in financial status and insurance coverage can limit ability to seek dental care. Lower income older adults with poorer oral health do not seek dental services in the US as often as their higher income counterparts (53). Dental services are not covered as part of traditional Medicare, the health insurance program for older adults in the US.

### 4.1 Limitations and strengths

Limitations include the smaller sample size when only including participants who are in both the 2018 HRS-LB and HRS-Dental Modules, and lack of clinical oral health status indicators. However, inclusion of the HRS-Dental Module allowed for capitalizing on recent available dental-specific variables and assessing individual’s perceptions of their oral health status and impact of overall oral conditions. Importantly, despite being a convenience subsample, it appeared similar in demographic characteristics to the merged 2018 HRS-CORE, HRS-LB and HRS-Dental Module sample in the larger latent class analysis sample of 4,703 (28). This analysis utilized cross-sectional data, so causality cannot be inferred. Future research can explore these findings further longitudinally, which would be facilitated by the inclusion of dental questions in the HRS-CORE rather than periodic experimental modules. Further, psychosocial analysis will only be possible when the HRS-LB subsample years also align. As psychosocial measures reflect self-reported perceptions, they can be affected by social desirability and recall biases. Loneliness especially tends to be stigmatized, and may be underreported.

Despite these limitations, the study’s strengths include rich characterization of loneliness among older adults using the validated UCLA 11-item measure. Clear patterns of risk and resource factors, also measured with validated instruments, were described in this analysis. This paper is an important contribution to the relationships between loneliness and oral health. Strengths include the richness and representativeness of the HRS national data and the combination of concepts of self-rated oral health and oral health-related quality of life and multiple aspects of loneliness.

## 5 Conclusion

Loneliness was a defining characteristic distinguishing the latent classes and associated with more risk factors and worse outcomes. Loneliness, life satisfaction, perceived age, social status, control, mastery, and chronic stressors vary widely for older adults and matter for oral health. Lonely older adults, whether they were satisfied with life or not, had worse outcomes. More psychosocial stressors and fewer resources were associated with worse SROH status and worse OHQOL scores. It is important for oral health providers to identify patients who are lonely to provide oral health interventions and referral for psychosocial interventions. Conversely, other providers who identify patients who are lonely may need referral for oral health care.

## Data availability statement

Publicly available datasets were analyzed in this study. This data can be found here: https://hrs.isr.umich.edu/data-products (the Health and Retirement Study website).

## Ethics statement

Ethical approval was not required for the study involving humans in accordance with the local legislation and institutional requirements. Written informed consent to participate in this study was not required from the participants or the participants’ legal guardians/next of kin in accordance with the national legislation and the institutional requirements.

## Author contributions

TF: Conceptualization, Funding acquisition, Investigation, Visualization, Writing – original draft, Writing – review & editing. KM: Conceptualization, Data curation, Formal analysis, Funding acquisition, Investigation, Methodology, Visualization, Writing – review & editing. JJ: Conceptualization, Funding acquisition, Investigation, Writing – review & editing. JP: Conceptualization, Funding acquisition, Investigation, Methodology, Visualization, Writing – review & editing. JW: Conceptualization, Funding acquisition, Investigation, Project administration, Writing – review & editing.
