# Corrigendum: Loneliness and low life satisfaction associated with older adults' poor oral health

**DOI:** 10.3389/fpubh.2025.1583484

**Published:** 2025-04-14

**Authors:** T. L. Finlayson, K. L. Moss, J. A. Jones, J. S. Preisser, J. A. Weintraub

**Affiliations:** ^1^Health Management and Policy, San Diego State University School of Public Health, San Diego, CA, United States; ^2^Department of Biostatistics and Health Data Science, Adams School of Dentistry, University of North Carolina at Chapel Hill, Chapel Hill, NC, United States; ^3^University of Detroit Mercy, Detroit, MI, United States; ^4^Department of Biostatistics, Gillings School of Global Public Health, University of North Carolina at Chapel Hill, Chapel Hill, NC, United States; ^5^Department of Pediatric Dentistry and Dental Public Health, Adams School of Dentistry, University of North Carolina at Chapel Hill, Chapel Hill, NC, United States

**Keywords:** loneliness, life satisfaction, oral health, quality of life, latent class analysis, psychosocial, older adults

In the published article, there was an error in Table 1 as published. Scores for the Life Satisfaction Domain-Specific scale were omitted. The corrected Table 1 and its caption appear below.

**Table 1:** Demographic and psychosocial characteristics, by self-rated oral health status and oral health quality of life (OHQOL), HRS 2018 dental module sample (*n* = 416).

	***N* = 416**	**Excellent or very good or good *N* = 301**	**Fair or poor *N* = 115**	**Mean (SD) OHQOL^c^**
**Demographics**	***N* (%)**	***N* (%)**	***N* (%)**	**Mean (SD)**
Race				
Caucasian	292 (70.9%)	226 (75.8%)	66 (57.9%)	9.22 (15.7)
African American	51 (12.4%)	27 (9.1%)	24 (21.1%)	18.0 (21.4)
Hispanic	44 (10.7%)	25 (8.4%)	19 (16.7%)	18.0 (22.5)
Other	25 (6.1%)	20 (6.7%)	5 (4.4%)	13.7 (17.0)
Sex				
Female	250 (60.1%)	183 (60.8%)	67 (58.3%)	12.0 (18.2)
Male	166 (39.9%)	118 (39.2%)	48 (41.7%)	10.5 (16.8)
Birth cohort				
AHEAD & CODA	13 (3.1%)	11 (3.7%)	2 (1.7%)	7.1 (12.0)
HRS original	88 (21.2%)	60 (19.9%)	28 (24.4%)	13.1 (17.8)
War babies	59 (14.2%)	41 (13.6%)	18 (15.7%)	9.0 (11.3)
Baby boomers	256 (61.5%)	189 (62.8%)	67 (58.3%)	11.6 (19.0)
Education				
<High school	46 (11.1%)	24 (8.0%)	22 (19.1%)	21.4 (21.8)
High school or equivalent	245 (58.9%)	166 (55.2%)	79 (68.7%)	13.0 (19.1)
College +	125 (30.1%)	111 (36.9%)	14 (12.2%)	4.7 (8.4)
Marital status				
Married	247 (59.7%)	189 (63.0%)	56 (49.1%)	8.8 (14.6)
Not married	167 (40.3%)	111 (37.0%)	58 (50.9%)	15.2 (20.8)
Live alone				
Yes	87 (20.9%)	62 (20.6%)	25 (21.7%)	12.5 (20.0)
No	329 (79.1%)	239 (79.4%)	90 (78.3%)	11.1 (17.0)
Household net wealth				
<$50,000–$50,000	107 (25.7%)	59 (19.6%)	48 (41.7%)	22.7 (24.5)
>$50,000–$200,000	81 (19.5%)	52 (17.3%)	29 (25.2%)	9.9 (13.2)
>$200,000–$500,000	91 (21.9%)	77 (25.6%)	14 (12.2%)	7.0 (11.9)
$500,000+	137 (32.9%)	113 (37.5%)	24 (20.9%)	6.4 (12.2)
Medicaid				
Yes	52 (12.5%)	28 (9.3%)	24 (20.9%)	25.3 (27.0)
No	363 (87.5%)	272 (90.7%)	91 (79.1%)	9.5 (14.9)
Location				
Urban	214 (51.8%)	154 (51.5%)	60 (52.6%)	11.8 (18.5)
Suburban	91 (22.0%)	70 (23.4%)	21 (18.4%)	12.6 (19.8)
Ex-urban	108 (26.2%)	75 (25.1%)	33 (29.0%)	9.5 (13.6)
Current smoker				
Yes	46 (11.1%)	23 (7.7%)	23 (20.0%)	24.1 (21.6)
No	368 (88.9%)	276 (92.3%)	92 (80.0%)	9.9 (16.5)
Current drinker				
Yes	249 (60.3%)	195 (65.4%)	54 (47.0%)	14.9 (20.2)
No	164 (39.7%)	103 (34.6%)	61 (53.0%)	9.2 (15.5)
Diabetes				
Yes	110 (26.6%)	66 (22.1%)	44 (38.6%)	16.1 (21.3)
No	303 (73.4%)	233 (77.9%)	70 (61.4%)	9.7 (15.9)
**Psychosocial characteristics**	**Mean (SD)**			
Lonely scale, mean (SD)^a^	1.52 (0.44)	1.46 (0.41)	1.69 (0.48)	0.30^d^
Life satisfaction wellbeing scale, mean (SD)^b^	5.10 (1.59)	5.35 (1.47)	4.46 (1.70)	−0.32^d^
Life satisfaction domain specific scale, mean (SD)^b^	3.67 (0.77)	3.81 (0.70)	3.29 (0.81)	−0.32
Perceived age scale, mean (SD)^b^	3.99 (1.04)	4.13 (0.99)	3.62 (1.10)	−0.28^d^
Feel Older				
Yes	51 (12.6%)	31 (10.4%)	20 (18.7%)	18.3 (19.1)
No	354 (87.4)	267 (89.6%)	87 (81.3%)	10.5 (17.4)
Constraints scale, mean (SD)^a^	2.06 (1.13)	1.85 (0.99)	2.63 (1.27)	0.25d^d^
Mastery scale, mean (SD)^b^	4.78 (1.14)	4.87 (1.15)	4.55 (1.09)	−0.09^d^
Perceived change in social status, mean (SD)^b^	6.63 (1.79)	7.00 (1.56)	5.63 (1.99)	−0.28^d^
Moved in social status				
Up	72 (17.5%)	53 (17.8%)	19 (16.7%)	9.62 (14.8)
Down	35 (8.5%)	21 (7.1%)	14 (12.3%)	19.0 (21.3)
No change	305 (74.0%)	224 (75.2%)	81 (71.1%)	11.0 (17.7)
Control domain^b^				
Over health	7.70 (2.07)	7.94 (1.87)	7.08 (2.40)	−0.17^d^
Over social life	8.20 (2.01)	8.38 (1.82)	7.72 (2.39)	−0.22^d^
Over financial situation	7.66 (2.33)	7.94 (2.13)	6.94 (2.64)	−0.12^d^
Lifestyle (% upsetting)				
Self-health problems	150 (36.1%)	92 (30.6%)	58 (50.4%)	17.3 (19.8)
Phy/Emot problems in SP/child	135 (32.5%)	96 (31.9%)	39 (33.9%)	14.8 (20.1)
Drug/alcohol probs fam member	52 (12.5%)	31 (10.3%)	21 (18.3%)	22.3 (23.1)
Financial strain	92 (22.1%)	54 (17.9%)	38 (33.0%)	18.4 (22.3)
Housing problems	30 (7.2%)	17 (5.7%)	13 (11.3%)	23.5 (19.7)
Problems in relationship	64 (15.4%)	48 (16.0%)	16 (13.9%)	12.8 (17.5)
Reg help ailing friend/fam	49 (11.8%)	31 (10.3%)	18 (15.7%)	13.9 (20.6)

a Higher mean scores are worse (higher psychosocial risk) for these scales: loneliness, constraints.

b Higher mean scores are better (lower psychosocial risk; more psychosocial resources) for these scales: life satisfaction wellbeing, life satisfaction domain-specific, perceived age, mastery, change in social status, and control.

c OHQOL, oral health quality of life summary score, higher scores indicate worse OHQOL. OHQOL includes items related to avoid foods, difficult to relax, avoided going out, self-conscious, and pain.

d Pearson correlation.

In the published article, there was an error in Table 2 as published. Scores for the Life Satisfaction Domain-Specific scale were omitted. The corrected Table 2 and its caption appear below.

**Table 2:** Mean (SE) scale items by LCA class, HRS 2018 dental module sample (*n* = 416).

	**Class A: not lonely/satisfied *N* = 201**	**Class B: lonely/ satisfied *N* = 103**	**Class C: lonely/unsatisfied *N* = 112**	***p*–value**
**Demographics**				
Race				0.02
Caucasian	152 (76.4%)	69 (67.7%)	71 (64.0%)	
African American	24 (12.1%)	11 (10.8%)	16 (14.4%)	
Hispanic	14 (7.0%)	18 (17.7%)	12 (10.8%)	
Other	9 (4.5%)	4 (3.9%)	12 (10.8%)	
Sex				0.02
Female	132 (65.7%)	50 (48.5%)	68 (60.7%)	
Male	69 (34.3%)	53 (51.5%)	44 (39.3%)	
Birth cohort				0.07
AHEAD & CODA	9 (4.5%)	3 (2.9%)	1 (0.9%)	
HRS original	41 (20.4%)	25 (24.3%)	22 (19.6%)	
War babies	38 (18.9%)	9 (8.7%)	12 (10.7%)	
Baby boomers	113 (56.2%)	66 (64.1%)	77 (68.8%)	
Education				0.03
<High school	22 (11.0%)	11 (10.7%)	13 (11.6%)	
high school or equivalent	105 (52.2%)	63 (61.2%)	77 (68.8%)	
College +	74 (36.8%)	29 (28.2%)	22 (19.6%)	
Marital Status				0.006
Married	131 (65.8%)	63 (61.2%)	53 (47.3%)	
Not married	68 (34.2%)	40 (38.8%)	59 (52.7%)	
Live alone				0.049
Yes	39 (19.4%)	16 (15.5%)	32 (28.6%)	
No	162 (80.6%)	87 (84.5%)	80 (71.4%)	
Household net wealth				<0.0001
<$50,000–$50,000	41 (20.4%)	22 (21.4%)	44 (39.3%)	
>$50,000–$200,000	34 (16.9%)	17 (16.5%)	30 (26.8%)	
>$200,000–$500,000	47 (23.4%)	23 (22.3%)	21 (18.8%)	
$500,000+	79 (39.3%)	41 (39.8%)	17 (15.2%)	
Medicaid				0.36
Yes	21 (10.5%)	13 (12.6%)	18 (16.1%)	
No	179 (89.5%)	90 (87.4%)	94 (83.9%)	
Location				0.79
Urban	107 (53.8%)	53 (51.5%)	54 (48.7%)	
Suburban	44 (22.1%)	24 (23.3%)	23 (20.7%)	
Ex-urban	48 (24.1%)	26 (25.2%)	34 (30.6%)	
Current smoker				0.009
Yes	16 (8.0%)	9 (8.8%)	21 (18.9%)	
No	185 (92.0%)	93 (91.2%)	90 (81.1%)	
Current drinker				0.10
Yes	130 (65.7%)	58 (56.3%)	61 (54.5%)	
No	68 (34.3%)	45 (43.7%)	51 (45.4%)	
Diabetes				0.20
Yes	46 (23.1%)	28 (27.2%)	36 (32.4%)	
No	153 (76.9%)	75 (72.8%)	75 (67.6%)	
**Psychosocial characteristics**				
Lonely scale, mean (SD)^a^	1.17 (0.16)	1.77 (1.77)	1.91 (0.43)	<0.0001
Life satisfaction wellbeing scale, mean (SD)^b^	5.94 (1.15)	5.36 (1.05)	3.35 (1.26)	<0.0001
Life satisfaction domain-specific scale, mean (SD)^b^	4.01 (0.57)	3.63 (0.57)	2.92 (0.63)	<0.0001
Perceived age scale, mean (SD)^b^	4.54 (0.85)	3.90 (0.78)	3.08 (0.90)	<0.0001
Feel older (yes)	7 (3.6%)	9 (8.7%)	35 (32.4%)	
No	187 (96.4%)	94 (91.3%)	73 (67.6%)	<0.0001
Constraints scale, mean (SD)^a^	1.51 (0.78)	2.15 (1.02)	2.97 (1.15)	<0.0001
Mastery scale, mean (SD)^b^	5.12 (1.05)	4.95 (0.86)	4.01 (1.16)	<0.0001
Perceived change in social status, mean (SD)^b^	7.31 (1.38)	6.86 (1.68)	5.20 (1.75)	<0.0001
Moved in social status				<0.0001
Up	38 (19.1%)	24 (23.5%)	10 (9.0%)	
Down	4 (2.0%)	3 (2.9%)	28 (25.2%)	
No change	157 (78.9%)	75 (73.5%)	73 (65.8%)	
Control domain, mean (SD)^b^				
Over health	8.18 (1.66)	8.03 (1.73)	6.55 (2.52)	<0.0001
Over social Life	9.03 (1.18)	8.07 (1.89)	6.81 (2.48)	<0.0001
Over financial situation	8.48 (1.62)	8.03 (1.84)	5.86 (2.79)	<0.0001
Lifestyle (% upsetting)				
Self-health problems	41 (20.4%)	32 (31.1%)	77 (68.8%)	<0.0001
Phy/emot problems in SP/child	42 (20.9%)	28 (27.2%)	65 (58.0%)	<0.0001
Drug/alcohol probs fam member	12 (6.0%)	13 (12.6%)	27 (24.1%)	<0.0001
Financial strain	11 (5.5%)	10 (9.7%)	71 (63.4%)	<0.0001
Housing problems	4 (2.0%)	3 (2.9%)	23 (20.5%)	<0.0001
Problems in relationship	10 (5.0%)	14 (13.6%)	40 (35.7%)	<0.0001
Reg help ailing friend/fam	14 (7.0%)	8 (7.8%)	27 (24.1%)	<0.0001

a Higher mean scores are worse (higher psychosocial risk) for these scales: loneliness, constraints.

b Higher mean scores are better (lower psychosocial risk; more psychosocial resources) for these scales: life satisfaction wellbeing, life satisfaction domain-specific, perceived age, mastery, change in social status, and control.

The authors apologize for this error and state that this does not change the scientific conclusions of the article in any way. The original article has been updated.

